# Complement System Part II: Role in Immunity

**DOI:** 10.3389/fimmu.2015.00257

**Published:** 2015-05-26

**Authors:** Nicolas S. Merle, Remi Noe, Lise Halbwachs-Mecarelli, Veronique Fremeaux-Bacchi, Lubka T. Roumenina

**Affiliations:** ^1^UMRS 1138, Centre de Recherche des Cordeliers, INSERM, Paris, France; ^2^UMRS 1138, Centre de Recherche des Cordeliers, Sorbonne Paris Cité, Université Paris Descartes, Paris, France; ^3^UMRS 1138, Centre de Recherche des Cordeliers, Sorbonne Universités, UPMC Université Paris 06, Paris, France; ^4^Ecole Pratique des Hautes Études (EPHE), Paris, France; ^5^Service d’Immunologie Biologique, Assistance Publique-Hôpitaux de Paris, Hôpital Européen Georges-Pompidou, Paris, France

**Keywords:** complement system, adaptive immunity, crosstalk TLR-complement, pathogen strategies for immune evasion, complement in cancer, complement and innate immunity, complement-related diseases, anaphylatoxins

## Abstract

The complement system has been considered for a long time as a simple lytic cascade, aimed to kill bacteria infecting the host organism. Nowadays, this vision has changed and it is well accepted that complement is a complex innate immune surveillance system, playing a key role in host homeostasis, inflammation, and in the defense against pathogens. This review discusses recent advances in the understanding of the role of complement in physiology and pathology. It starts with a description of complement contribution to the normal physiology (homeostasis) of a healthy organism, including the silent clearance of apoptotic cells and maintenance of cell survival. In pathology, complement can be a friend or a foe. It acts as a friend in the defense against pathogens, by inducing opsonization and a direct killing by C5b–9 membrane attack complex and by triggering inflammatory responses with the anaphylatoxins C3a and C5a. Opsonization plays also a major role in the mounting of an adaptive immune response, involving antigen presenting cells, T-, and B-lymphocytes. Nevertheless, it can be also an enemy, when pathogens hijack complement regulators to protect themselves from the immune system. Inadequate complement activation becomes a disease cause, as in atypical hemolytic uremic syndrome, C3 glomerulopathies, and systemic lupus erythematosus. Age-related macular degeneration and cancer will be described as examples showing that complement contributes to a large variety of conditions, far exceeding the classical examples of diseases associated with complement deficiencies. Finally, we discuss complement as a therapeutic target.

## The Complement System

**Figure 1:**
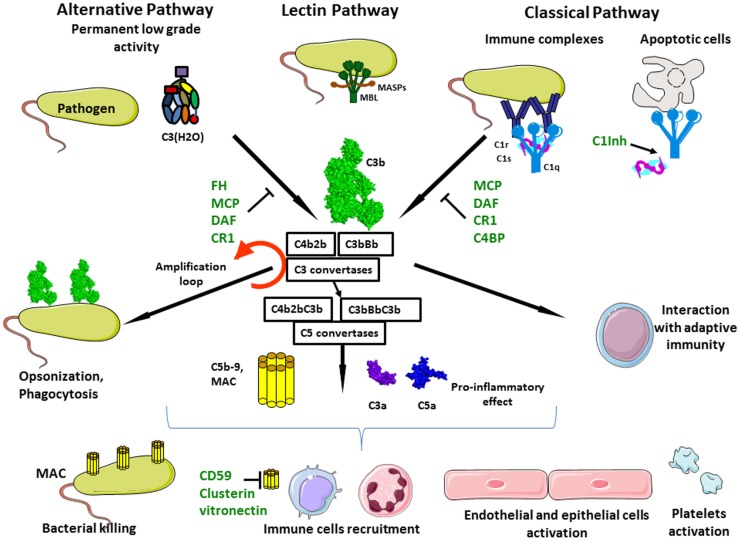
**Complement activation**. Complement system is composed of three different pathways. CP is activated by immune complex formation on pathogen surface and by calreticulin expressed on apoptotic cells, leading to C1 complex association. LP recognizes mannose-terminating glycan on pathogens leading to MBL MASP complex activation. Both induce formation of the classical C3 convertase C4b2a. AP is permanently activated at low level by spontaneous hydrolysis of C3 into C3(H_2_O). Lack of complement inhibitor on pathogens induces alternative C3 convertase activation C3bBb. Complement activation leads to opsonization and phagocytosis by C3b deposition, bacterial lysis by C5b–9 complex formation and inflammation by recruitment of immune cells, endothelial and epithelial cells activation, and platelets activation.

Complement system represents a major part of the innate immunity. It is a cascade of soluble proteins and membrane expressed receptors and regulators (Figure 1), which operates in plasma, in tissues, on cell surface, and even within the cell. It is composed of more than 40 proteins, the soluble ones being produced mainly by the liver. Complement was discovered at the end of the nineteenth century and described as a “factor” or “principle” capable to induce bacterial lysis. After that, for a long time, complement system has been considered as a supportive part of the innate immunity and received relatively little attention from the immunologists. Over the years, it became clear that complement has versatile functions and that its action extends far beyond the simple bactericidal activity. In a healthy individual, it orchestrates the immunologically silent clearance of host cells after their programed cell death. Complement cascade is activated immediately after encountering the pathogen. Hence, complement participates in pathogen opsonization, tagging it for engulfment by antigen presenting cells (APC); it plays a central role in the inflammatory process and modulates the activity of T- and B-cells. After generation of pathogen-specific antibodies, complement contributes in the clearance of immune complexes and pathogen elimination. Studies over the years demonstrated that complement takes part in nearly every step of the immune reaction and that it deserves a central position in the immunological research. Unfortunately, the lack of coherence in complement proteins nomenclature and the complexity of the enzymatic cascade render complement one of the “most complicated and incomprehensible” parts of immunology and is frequently avoided by students and scientists. With this review, we aim to underline the crucial importance of complement in physiology and pathology.

## Complement in Physiology

### Sampling for foreign and altered cells

**Figure 2:**
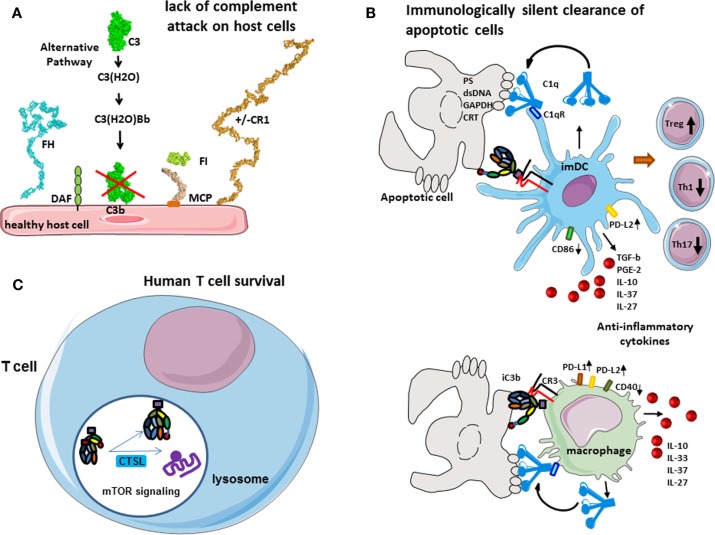
**Role of complement in physiology**. **(A)** Protection of host cells against complement. Complement AP is permanently activated and deposits C3b molecules to any surface. On host cells, these C3b molecules are rapidly inactivated by different membrane expressed or plasma complement regulators. **(B)** Immunologically silent clearance of apoptotic cells. Limited complement activation occurs on apoptotic cells. C1q recognizes “eat-me” signals on the surface of dying cells. It serves as a bridging molecule, facilitating the phagocytosis by dendritic cells or macrophages. Uptake of C1q-opsinized cargo induces an anti-inflammatory program, increased expression of immunological checkpoint molecules, and prevents up-regulation of maturation markers. iC3b on apoptotic cells interacts with CR3 on phagocytes and induces anti-inflammatory response. **(C)** Complement in human T cell homeostasis. Cathepsin L cleaves C3 intracellularly, generating C3a and C3b. C3a binds on C3aR, expressed in the lysosomes, and stimulates mTOR signaling pathway. This signaling is important for the cell survival in resting state.

The main complement rule is that everything that is not specifically protected has to be attacked. Host cells carry an armamentarium of “don’t attack me” molecules, which are either expressed by the cell or recruited to the cell membrane from the plasma. Therefore, any cell, debris, microorganism, or artificial material lacking these molecules (and carrying −OH or −NH_2_ chemical groups, which is the case of all biological and most synthetic materials) will represent an “activating surface” and will support complement deposition, i.e., covalent binding of C3b, an activated form of the central complement component C3. This action is provided by the so-called alternative complement pathway, which is permanently active and constantly probes the environment for the presence of activating surfaces (Figure 2A). In addition, several pattern recognition molecules recognize material that has to be eliminated, such as apoptotic cells, debris, pathogens, immune complexes, and activate the classical and lectin complement pathways (CP and LP) (Figure 2B). All pathways converge at the point of C3 cleavage, which results in generation of bioactive fragments C3a and C3b. C3b binds covalently to any surface and, if it is not specifically protected. The level of C3 deposition is increased by the so-called “amplification loop” of the alternative pathway (AP). Detailed description of the mechanisms of activation and regulation of the complement system are described in “Complement system part I – molecular mechanisms of activation and regulation” (1), from the same research topic of Frontiers in Immunology.

Briefly, in a healthy organism, the sentinel role is assured by the permanent activity of the AP (1 and 2). A number of complement components are produced in biologically inactive form, which allows them to co-exist in plasma, or to be produced by the same cells, without interacting with one another and inducing unnecessary and undesired complement activation. Complement components are activated in a cascade fashion after a triggering event, each step of the chain reaction, resulting in a conformational change or a cleavage of the downstream component, which becomes activated and gains the capacity to activate the subsequent component in the cascade. Spontaneous hydrolysis, called tick-over, of C3 plays an important role in the immune surveillance and AP activation (2). C3 is present in plasma at high concentrations (~1 mg/ml) and a small portion of it undergoes spontaneous hydrolysis of a particular thioester bond between the side chains of two aminoacids, located in the thioester domain (TED). This hydrolysis induces a dramatic conformational change in C3 and renders it biologically active due to exposure of novel binding sites. In this new form – the C3(H_2_O), it recruits two other plasma molecules – factor B (FB) and factor D (FD). FD cleaves C3(H_2_O)-bound FB to generate an enzymatic complex C3(H_2_O)Bb, called fluid phase C3 convertase. The name convertase indicates that this enzymatic complex can cleave (convert) a native C3 molecule into bioactive fragments C3a (the small fragment) and C3b (the big fragment). C3a is an anaphylatoxin – a pro-inflammatory molecule, which activates the surrounding cells when reaching a threshold concentration. Upon releasing the C3 ANA domain, which becomes the C3a molecule, the remaining part C3b undergoes a dramatic conformational change, similar to that of C3(H_2_O). In the newly generated C3b, contrary to C3(H_2_O), the thioester bond is not hydrolyzed but becomes transiently exposed, allowing, for a very short time, a covalent reaction with OH^−^ or NH2^−^ groups on any molecule or cell in its immediate surroundings. If this bond is not formed, the very short-lived nascent form is hydrolyzed and inactivated in few milliseconds, leaving the inactivated C3b molecule in the fluid phase. When covalently bound to a cell, C3b has different fates depending if it is on a host cell or on a foreign surface.

### Protection of the host cells against complement attack

The deposition of C3b is highly controlled on host cells. Host cells express complement regulatory molecules on their surface (membrane bound such as membrane cofactor protein, MCP, CD46 or complement receptor 1, CR1, CD35), or recruit plasma regulators, like factor H (FH), which bind to C3b. These proteins serve as cofactors, allowing interaction with a plasma serine protease factor I (FI), which cleaves C3b into iC3b (Figure 2A). This results in a conformational change, which suppresses the ability to interact with FB but exposes of novel binding sites in the iC3b molecule, allowing it to interact with other complement molecules. FH also blocks the binding of FB and FD to C3b, thus preventing the formation of C3 convertases. The overall action of all these regulators results in the prevention of complement activation on host cells. In the absence of surface regulators, such as in the case of pathogens or other foreign surface, C3b interacts with FB and FD, forms C3 convertases, which cleave more molecules of C3 to C3a and C3b, thus fueling the amplification loop and allowing full-blown complement activation and, finally, pathogen elimination.

### Immunologically silent phagocytosis of apoptotic cells

Between these two extremes (healthy cell and pathogen) remains the case of stressed and apoptotic host cells. The human body is composed to a myriad of different cells, forming a highly organized system. The proliferation, differentiation, activity, and also the death of cells in this system are tightly controlled. Programed cell death induces major cellular modifications. During a classical cell death, the cell surface undergoes many structural and molecular modification, leading to “eat-me” signals expression. Phagocytes recognize these signals and execute the degradation of apoptotic cell without mounting of an immunologic response. Among these modifications, a major one is the expression of phosphatidylserine (PS) on the external side of the cell membrane, which is normally sequestered in the inner surface of the cell membrane (3). Also, the expression level of some complement regulators (such as MCP) can be reduced. Clearance of apoptotic cells is critical for many physiological processes, including development, tissue remodeling, and maintenance of homeostasis.

The complement system plays a major role in the tolerogenic perception of apoptosis, which is in part mediated by opsonization with C1q and iC3b and subsequent clearance of dying cells (4, 5) (Figure 2B). C1q, which is the recognition molecule of the CP, is produced by macrophages and dendritic cells (DCs) and binds to a variety of ligands that can be expressed on the surface of apoptotic cells such as PS, double stranded DNA, GAPDH, or calreticulin (6–9). C1q has complex immune-modulatory effects and a failure of C1q to opsonize apoptotic cells results in defective phagocytosis by monocytes (10) and activation of the DCs (11). Consistently, a quantitative or functional deficiency in C1q may be related to improper apoptotic cell clearance and autoimmunity (12, 13). Similar functions are described for mannose binding lectin (MBL), one of the recognition molecules of the LP (14).

C1q coated apoptotic cells suppress macrophage inflammation through induction of interleukine 10 (IL-10), IL-27, IL-33, and IL-37, inhibit inflammasome activation and increase the expression of negative regulators ASC2 and NLRP12 (15). It has recently been demonstrated that opsonization of apoptotic cells by C1q induced an increase of the expression of PD-L1 and PD-L2 and a diminution of CD40 at the surface of macrophages (16). Similar effects are observed also on dendritic cells. Presentation of self-antigens by DC in the presence of C1q promotes the development of regulatory T cells (Treg) and the production of anti-inflammatory cytokines such as TGF-b, IL-10, PGE2, IL-37, and IL-27 and thus confers tolerance. In addition, opsonization of the apoptotic cells with C1q induced a high expression of PD-L2 and less CD86 on dendritic cells surface after phagocytosis. This “polarization” by C1q-induced decrease of T helper 1 (Th1) and Th17 and proliferation. The non-maturation of the phagocytes, which is showed by the expression of CD40 and CD86 on macrophages and dendritic cells, respectively, and by the secretion of anti-inflammatory cytokines, makes the phagocytosis immunologically silent (16). Therefore, C1q is of critical importance for the silent, non-immunogenic clearance of apoptotic cells (17) (Figure 2B).

The inactivated fragment of the central complement component C3–iC3b participates in the clearance of apoptotic cells via interaction with CR3 on monocytes, macrophages, DC, and microglial cells (18–20). iC3b opsonization and CR3-dependent phagocytosis is accompanied by a down-regulation of the pro-inflammatory mediator IL-12 and a lack of oxidative burst in macrophages (21, 22) or by a reduction in the expression of costimulatory molecules and impaired maturation of DC (23, 24). This confers anti-inflammatory properties and supports tolerogenic apoptotic cell clearance.

### Cell homeostasis

In the native state, different cell types secrete complement components and generate C3a and C5a in their microenvironment, an important fact for their viability and function. Liszewski et al. demonstrated that C3 activation can occur continuously within human T cells, mediated by cathepsin L (CTSL) and in a C3 convertase-independent manner (25) (Figure 2C). In resting T cells, this C3a binds to intracellular C3aR, expressed in lysosomes, but not on the cell surface. Intracellular C3aR signaling sustains basal mTOR activation (25), required for homeostatic cell survival (26). Of note, C3a generated outside the cell cannot restore the mTOR signaling in cells with inhibited CTSL, suggesting the importance of intracellular generated C3a (25). Upon T cell receptor (TCR) activation, C3aR is expressed on the cell surface and contributes to the mounting of Th1 response in concert with CD46 signaling.

Of note, the phenomenon of intracellular C3 cleavage seems to be species specific, because it does not operate in mice. In mice, resting T cells synthesize complement components constitutively, complement activation occurs in their microenvironment, and the resultant C5a and C3a signaling, through the C5aR and C3aR, participates in cell viability by maintaining the level of phosphorylated AKT (PKB), a T cell activation intermediate that suppresses apoptosis (27). In the absence or after blockade of C5aR and C3aR, the expression of MHC class II and costimulatory-molecules is decreased on dendritic cells (27, 28). The exact contribution to T cells survival of the intracellular and extracellular C3a generation and C3aR signaling is still not well defined. It is important to note that AKT (PKB) and mTOR belong to the same signaling cascade, which has anti-apoptotic, survival, and proliferation effects (29). The intracellular complement activation is not restricted to T cells (25), and may have an important role in the physiology of other human cell types.

## Complement as a First Line of Defense Against Pathogens

### Direct killing

**Figure 3:**
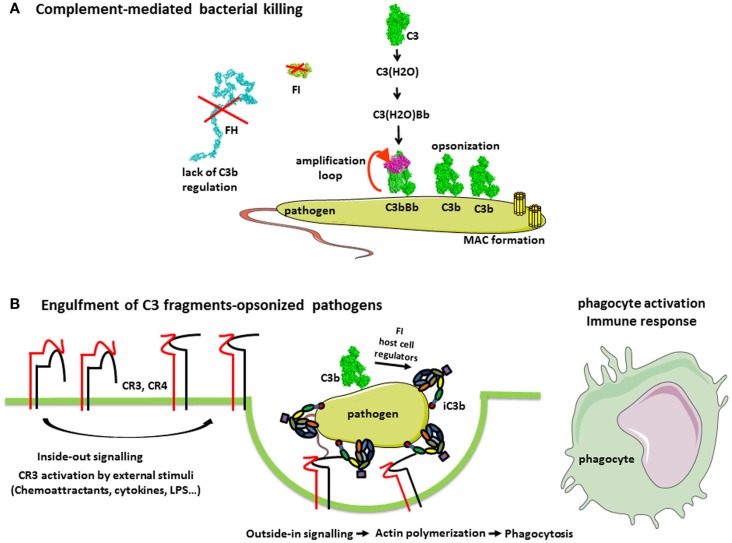
**Complement in the defense against pathogens**. **(A)** Complement-mediated bacterial killing. C3b is deposited on any pathogen surfaces due to the constant activity of the AP. Since most pathogens do not have complement regulatory molecules, C3b is not inactivated and interacts with FB and FD to form a C3 convertase C3bBb. This enzymatic complex cleaves more C3 molecules, resulting in pathogen opsonization with C3b. Further, the cascade proceeds to a C5 convertase and MAC formation, contributing to bacterial killing. **(B)** Complement receptors-mediated phagocytosis of C3b and iC3b-opsonized pathogens. Extracellular stimulatory signals, which are necessary for the CR3-mediated phagocytosis, include chemoattractants (not only chemokines but also bacterial formylpeptides and C5a for neutrophils), cytokines (e.g., TNF-α), and bacterial products (e.g., lipopolysaccharide). External stimuli activate the integrin CR3, (i.e., change to a conformation with high affinity for iC3b) by a Rap-1-mediated signaling. Stabilization of CR3 high-affinity conformation by its engagement with iC3b triggers a RhoA-mediated signaling, which drives the actin polymerization to engulf iC3b-coated target. Complex actin movements are then involved in the intracellular movement of the phagosome during its maturation to the phagolysosome.

Pathogens are attacked by all complement pathways, but the prevalence of one or another pathway depends on the exact membrane composition. C3b generated by the spontaneous activation of the AP tag all pathogens and, when no regulator is present, the cascade and the deposition of C3b are accelerated (Figure 3A). In addition, the pathogen-associated molecular patterns can be recognized by the recognition molecules of the CP and LP C1q and MBL or ficolins. C1q recognizes mostly charged patterns and can bind to more than 100 different target molecules (30), including pathogen-associated molecular patterns such as lipopolysaccharide (LPS) (31) or bacterial porins (32). MBL binds to a wide range of repeating sugar arrays normally presented by many microorganisms, including mannose structures on fungal and micrococcal surfaces, and *N*-acetylglucosamine residues in cell walls of bacteria, in order to initiate neutralization of these organisms (33). C1q may bind also on natural antibodies, which due to their polyreactivity can recognize the pathogens (34). Upon target recognition, C1q undergoes conformational change and activates the two serine proteases C1r and C1s, associated with it in the context of the C1 complex (35, 36). Despite the similarity between C1 and MBL/MASP complex architectures, the mechanism of activation of the CP and LP differ (37). In particular, the serine proteases of the LP, MASP-1, and 2, are associated with different MBL (or ficolin) molecules, thus requiring a juxtaposition to allow MASP-1 from one complex to activate MASP-2 from the adjacent complex (38, 39). However, MASP-2 alone provides about 10% to cleave its natural substrate C4 by auto-activation (40). Activated serine proteases of the CP and LP cleave C4 and C2 to allow formation of the CP C3 convertase C4b2a, which cleaves C3. If this convertase is not regulated, C3 deposition will be accelerated and the amplification loop will be turned on. Binding of an additional C3b molecule in the immediate proximity, or most probably on the C3 convertase itself, modifies the specificity of the enzyme (41). It then starts to cleave C5, thus turning on a C5 convertase of the classical (C4b2aC3b) or alternative (C3bBbC3b) pathway. Cleavage of C5 gives rise to a powerful anaphylatoxin C5a and to a C5b fragment, which initiates the terminal part of the cascade, common for all activation pathways.

Activation of the terminal complement pathway results in the formation of the membrane-attack complexes (MAC), which forms large, 10 nm wide, pores in the target membrane (42). In fact, most pathogens are able to repair MAC-induced lesions and are resistant to complement lysis by MAC, as Gram-positive bacteria (43). Nevertheless, some Gram-negative bacteria are relatively sensitive to complement killing, particularly the meningitis causing *Neisseria* species (44, 45). This is illustrated by the susceptibility of individuals, deficient in terminal complement components as well as in properdin, to recurrent meningitis. Gram-positive bacteria have a very thick cell wall, which MAC cannot penetrate, therefore being resistant to complement-mediated lysis. Metabolically active nucleated cells are also resistant to lysis by complement (46, 47). However, increase of Ca flux and signal transduction have been described as the result of the insertion of multiple MAC in the membrane, inducing either apoptosis and cell killing or leading to cell proliferation depending on the cell type (48, 49). The molecular mechanisms of complement activation on pathogens are reviewed in detail and illustrated in Merle et al. (1).

### Opsonization and phagocytosis

The main role of complement in pathogen elimination is indirect, namely, the deposition of complement fragments on the surface of pathogen targets, so-called opsonization that allows their recognition, ingestion, and destruction by phagocytic cells, neutrophils, monocytes, and macrophages (Figure 3B). Both IgG antibodies and C3 fragments are the classical opsonins. But complement opsonization, resulting from the direct activation of the AP on pathogens surface allows their elimination by phagocytes before the mounting of an adaptive immune response and the appearance of antibodies (Figure 3). Phagocytes express specific receptors for C3 fragments, described in “Complement system part I – molecular mechanisms of activation and regulation” (1).

CR1 is a complement component molecule (CCP) domain containing molecule, involved in the control of C3 convertases. It is present on erythrocytes, on phagocytes, and on kidney glomerular podocytes and binds C3b and C4b. CR1 on erythrocytes plays a major role in the clearance of soluble immune complexes, by transporting them to the liver and spleen, where they are cleared by macrophages. The binding of C3b-coated targets to phagocyte CR1 is not sufficient to trigger phagocytosis, but C3b–CR1 interaction enhances the FcγR-mediated phagocytosis of targets bearing both IgG and C3b. Moreover, immune mediators that activate phagocytes, such as fibronectin (50) or LPS (51), induce the phagocytosis of targets opsonized with C3b only. However, this is probably partially mediated by CR1 in that case, since elastase, a major protease released by activated phagocytes, is able both to degrade CR1 and to cleave C3b into iC3b, allowing then iC3b-coated targets to be recognized by the efficient phagocytic receptor CR3 (52).

CR3 and CR4 are specific receptors for iC3b, among C3 fragments, able to induce the phagocytosis of iC3b-coated targets (53, 54). CR3 and CR4 belong to the integrin family, involved in cell adhesion processes, due to their ability to interact, in particular, with intercellular molecule-1 (ICAM-1), present on many cells, including endothelial cells. Integrins are formed from two chains, alpha and beta bearing magnesium ions necessary for their function. CR3 (also called MAC-1, αMβ2 or CD11bCD18) and CR4 (p150,95, αxβ2 or CD11cCD18) form, with LFA-1 and αDβ2, the leukocyte-specific integrin subfamily sharing the β2 chain (CD18). The CR3 also bear a lectin site, different from the iC3b and ICAM-1 binding site, and able to recognize microorganism-derived sugar ligands. If both the lectin and the iC3b-binding sites of CR3 are engaged, the strength of the CR3-mediated phagocytic response is enhanced (55).

CR3 is present on monocytes and its expression is up-regulated upon monocyte to macrophage differentiation. Resting neutrophils express low levels of CR3 on their surface but these levels increase dramatically following cell activation, due to the externalization of large intra-granular pools of receptors. CR4 is expressed poorly on neutrophils and monocytes, but its expression increases upon monocyte differentiation to macrophage or to dendritic cells.

The cellular processes involved in the phagocytosis of IgG- or C3-opsonized targets are different. When phagocytosis is mediated primarily via CR3, the reaction is slow and pathogens gently sink into the phagocyte, by a process involving actin polymerization dependent on the small GTPase Rho (53). By contrast, the Fcγ receptors provide a very vigorous engulfment of IgG-coated pathogens, with membrane extensions driven by small GTPases Rac and Cdc42. However, the CR3-mediated phagocytic response to iC3b-coated targets is boosted by the participation of additional receptors such as toll-like receptors (TLRs) recognizing pathogen patterns.

Indeed, it is worth noting that CR3 and CR4 induce phagocytosis mainly in conjunction with stimuli, such as pro-inflammatory cytokines, which activate phagocytic cells (56). These stimuli induce an inside-out signaling, which switches integrins to an active conformation with a higher affinity for iC3b and ICAM-1 (53). This may represent a control mechanism to avoid unwanted responses to host cells passively coated with a few iC3b molecules resulting from the continuous low-grade activation of the AP.

Apart from CR3 and CR4, some resident macrophages such as liver Kupffer cells express CRIg, a receptor for iC3b belonging to the immunoglobulin family and able to mediate the phagocytosis of iC3b-coated pathogens (57).

The main consequence of phagocytosis is the elimination of pathogens. Internalized microorganisms are killed both by toxic reactive oxygen compounds, generated through a NADPH oxidase complex assembled at the phagosomal membrane, and by microbicidal components, such as lysozyme and proteases, present in phagocyte granules fused with the phagosome to form the phagolysosome. Finally, CR3-mediated phagocytosis results in the apoptosis of phagocytic cells, an important step of the resolution of infection and inflammation (58).

## Complement in Inflammation

### Anaphylatoxins and their receptors

**Figure 4:**
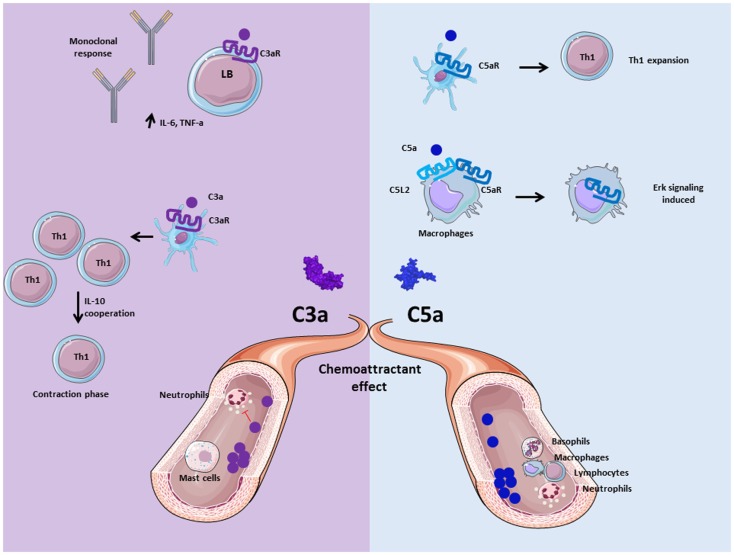
**Role of anaphylatoxins C3a and C5a**. Anaphylatoxins C3a and C5a participate in inflammation by interacting and activating immune cells via C3aR and C5aR, respectively. C3a is implicated in the adaptive immunity by inducing monoclonal response from B cells and up-regulation of pro- inflammatory cytokines. Moreover, C3a facilitates the contraction phase of T cells by increasing IL-10 synthesis. C5a is implicated in Th1 expansion to improve adaptive immunity response, and allows C5aR internalization in presence of C5L2 to induce ERK signalization and pro-inflammatory effect of macrophages. Both are chemoattractant molecule, and allow mast cells migration for C3a, basophils, macrophages, neutrophils, and lymphocytes recruitment for C5a at the inflammatory site. Nevertheless, C3a has an anti-inflammatory effect on neutrophils by inhibiting their degranulation and recruitment.

Complement anaphylatoxins C3a and C5a play a critical role in the modulation of immune system activity by complement. C3a and C5a are the small fragments released after cleavage of C3 and C5 by the C3 and C5 convertases of the classical and APs. They contribute to the inflammation and activate immune cells and non-myeloid cells, which express G-protein coupled anaphylatoxin receptors C3aR and C5aR (59, 60) (Figure 4). Anaphylatoxins stimulate inflammation by inducing an oxidative burst in macrophages (61), eosinophils (62), and neutrophils (63). Moreover, C3a and C5a induce histamine production by basophils (64) and mast cells (65), resulting in vasodilatation. Even if pro-inflammatory effects of C3a are not in question, studies highlight the anti-inflammatory role of C3a in different contexts (66). Neutrophil migration and degranulation are prevented by the presence of C3a, whether others granulocytes are activated by this anaphylatoxin (63, 67, 68). Thus, this suggests an anti-inflammatory role in acute phase of inflammation, and in cases of ischemia–reperfusion injury and in sepsis mouse models (69, 70).

The activation product of C4, C4a, seems also to have a functional activity on macrophages and monocytes (71, 72). Nevertheless, no C4a receptor has been reported, making it difficult to ascertain the physiological role of C4a (73). More studies are needed to understand whether C4a is an anapylatoxin and what is its mode of action.

In plasma, C3a and C5a are quickly converted by carboxypeptidase N and carboxypeptidase B into C3a-desArg and C5a-desArg, by cleavage of the C-terminal arginine (74–76). Recent study determined a central role of C3a in carboxypeptidase B2 (CBP2) negative mice, which are unable to convert C3a and C5a to C3a-desArg and C5a desArg (77). Using a treatment with blocking antibody against C3aR or C5aR, mice with only a functional C3a/C3aR axis present better survival after sepsis induction, whereas mice with only a functional C5a/C5aR axis present a less survival compare to wild-type mice. These data demonstrate two opposite effects between C3a and C5a, highlighting the complex role of C3a depending on the context. Another particularity of C3a and C5a is that C3a-desArg loses its ability to bind to C3aR and C5a-desArg has 90% weaker pro-inflammatory activity compared to C5a, as shown in human astrocyte model (78). By contrast, murine C5a-desArg is as potent as murine C5a upon binding to C5aR on murine cells (79). These inter-species differences have to be taken into account when *in vivo* experiments are analyzed.

Human *C3a* specifically binds its receptor *C3aR* (80), whereas C3a-desArg and C5a cannot bind to this receptor (81). C3aR belongs to the G-protein coupled receptor (GPCR) family with seven transmembrane domains. C3a binding leads to transduction of intracellular signals via heterotrimeric G-proteins and phosphorylation of PI3K, Akt, and mitogen-activated protein kinase (MAPK), leading to chemokine synthesis in human (82). Moreover, in human mast cells, C3a plays a role of chemoattractant molecule and can play an important role in hypersensitivity and inflammatory process (83). In case of chronic inflammation, C3a has pro-inflammatory activity and contributes to disease progression (60). In human monocytes and monocyte-derived macrophages, C3aR and TLR-4 costimulation induce the production of pro-inflammatory mediators, such as IL-1β, tumor necrosis factor alpha (TNF-α), IL-6, and PGE2 (84, 85). Contrary to these pro-inflammatory functions, in acute inflammation conditions C3a prevents mobilization and degranulation of neutrophils (66, 68). The difference in the response of inflamed tissues to C3a, between the acute and chronic phases of inflammation, may well be due to the differing cell types involved (e.g., neutrophils versus monocyte/macrophages) (66).

C3a modulates also the responses of the cells of the adaptive immunity. Human C3a has been described to regulate B cell function by suppressing the polyclonal immune response, IL-6 and TNFα release, in a dose-dependent manner (86). Mice C3aR signaling on DCs is important for the generation of Th1 cells (28, 87). A lack of C3aR activation on DC induces a Th2 polarization and favors the emergence of Treg. C3aR is also expressed on adaptive immune cells, such as T lymphocytes. Indeed, the importance of C3aR and C5aR expression for Th1 induction has been demonstrated in mice (27, 88, 89). Moreover, C3 deficiency induced a decreased expression, on T cells, of the α and β chains of the IL2 receptor. This may explain the aberrant Th1 response observed in patients with C3 deficiency. C3a also participates in the contraction phase of Th1 by regulating IL-10 expression (88).

C3aR is expressed not only by the immune cells but also by endothelial and epithelial cells. Stimulation of C3aR on endothelial cells induces a rapid mobilization of intracellular granules, Weibel–Palade bodies, containing von Willebrand factor and P-selectin. Hence, the cell acquire a pro-inflammatory and pro-thrombotic phenotype, since P-selectin helps the recruitment of leukocytes via binding to PSGL-1 (90) and von Willebrand factor mediates platelet adhesion (91). Moreover, human and mouse P-selectin binds to C3b on cell surfaces and serves as a platform for the formation of C3 convertases and the activation of the AP (92, 93). C3a activation of endothelial cells thus forms an amplification loop of complement activation, implicated in microvascular thrombosis, including in a mouse model of Shiga-toxin (Stx2)/LPS induced hemolytic uremic syndrome (HUS) (93).

Human and mouse *C5a* bind to *C5aR*, which also belongs also to the GPCR family (94). C5aR stimulation induces downstream effect such as activation of PI3K-γ (95, 96), phospholipase Cβ2 (97), phospholipase D (98), and Raf-1/B-Raf-mediated activation of MEK-1 (99). C5a is known to be a powerful chemoattractant molecule and plays a critical role in the inflammatory response by recruiting immune cells such as macrophages (100), neutrophils (101), basophils (102), and myeloid-derived suppressor cells (MDSCs) (103). Human C5a also recruits adaptive immune cells, such as T cells, which express constitutively C5aR on their surface (104). Human B cells express C5aR but respond to C5a only in case of activation (105). In a CD55^−/−^ mouse model, it has been demonstrated that C5a production by APC is essential for differentiation of T cell in Th1 effector cells (106). Indeed, addition of C5a on CD80^−/−^, CD86^−/−^, and CD40^−/−^ APC restored T cell activation, providing a link between GPCR, costimulation signals via CD28 and T cell survival (27). The axis C5a/C5aR appears to have a crucial role in sepsis, based on the observation that the inhibition of C5a/C5aR interaction decreased the mortality in a rat model (107). Another function of C5a is to induce vascular endothelial growth factor (VEGF) expression and to promote angiogenesis in a human retinal model (108). In a model of enteric infection, C5a is generated thanks to the stabilization of the AP C3 convertase by properdin and leads to IL-6 release from colonic epithelial cells (109). Then, IL-6 regulates inflammation induced by bacteria. In properdin deficient mice, the C5a-dependent IL-6 production is impaired, aggravating the infection. This provides evidences of the implication of C5a in the defense against bacteria-triggered epithelial injury.

*C3a, C5a, and C5a desArg* are also able to bind *C5L2*. In human, C3a-desArg is able to bind C5L2 and regulates triglyceride synthesis rate (110). C5a has a lower affinity for C5L2, as compared to C5a desArg in human basophil cell lineage (111). As C5a- and C3a-receptors, C5L2 is composed by seven transmembrane domains but it is not coupled with G protein (112). Previous work on human neutrophil determined a role of C5L2 as a negative regulator of anaphylatoxin receptor activity after activation and interaction with β-arrestin, this was confirmed in mouse model (113, 114). However, its role remains unclear. C5L2 has been described as an anti-inflammatory receptor, because C5L2^−/−^ mice have increased production of pro-inflammatory cytokines IL-6 and TNF-α (115). Nevertheless, deficiency of C5L2 on macrophages, neutrophils, and fibroblasts decreased their proinflammatory capacity *in vitro*. C5L2 is also essential for the C5a-mediated cell infiltration in *in vivo*, in a mouse model, suggesting a role of positive modulator of C5a-induced responses (67).

C5L2 expression in human atherosclerosis lesions is correlated with an increase of IL-1β and TNF-α release, supporting the pro-inflammatory effect of C5L2 (116). It has recently been reported that human C5L2 and C5aR are able to form a heterodimer (117). This complex induces internalization of C5aR upon C5a binding and promotes ERK and MEK signaling in a mouse model of acute experimental colitis (118). This internalization of GPCR has already been demonstrated in *in vitro* assays as essential for the induction of the late stage of ERK signaling (119, 120).

### Crosstalk between C3aR and C5aR signaling pathways with toll-like receptor signaling

**Figure 5:**
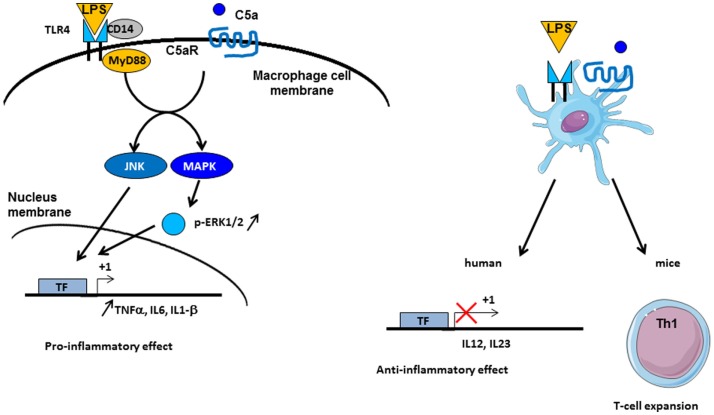
**Crosstalk between complement and TLR pathways**. C5a/C5aR signaling pathway can cooperate with TLR-4 activation by LPS on macrophages. Intermediate signaling pathways JNK and MAPK are activated and thus lead to pro-inflammatory effect by TNF-α, IL6, and IL1-β synthesis. On dendritic cells (DCs), TLR-4 and C5aR cooperate in different manner between mice and human. *In vivo* experiments have demonstrated an implication in Th1 cells expansion, whereas in human, an anti-inflammatory role of TLR-4/C5aR collaboration has been described by an antagonized effect on IL-12 and IL-23 synthesis by DC.

C3a and C5a are able to induce potent inflammatory pathways via their receptors C3aR and C5aR. The implication of intermediates such as NF-kB, MAPK, and c-Jun N-terminal kinase (JNK) in their transduction pathways suggests a potential crosstalk with other pathways, such as those of TLRs. Indeed, complement is involved in TLR-induced inflammation (121) (Figure 5). Co-activation of MyD88-dependent TLRs (TLR-2, TLR-4, and TLR-9) and complement in CD55^−/−^ knockout mice increased plasma inflammatory cytokines such as IL-6, TNF-α, and IL-1beta. Moreover, complement activation by the fluid phase cobra venom factor (CVF) synergizes with LPS to cause a dramatic increase of IL-6 production. These results suggest a strong interaction between TLRs and complement signaling *in vivo* to promote inflammation and modulate adaptive immunity (121). Complement may interact with TLR signaling by C3aR and C5aR, because of the involvement of MAPK, extracellular signal-regulated kinase (ERK1/2), and the JNK, but not p38 MAPK (121). TLR2 also crosstalks with CR3, since TLR2 can transactivate CR3 via PI3K by an inside-out signal (122). In turn, CR3 can regulate TLR2 signalization by recruiting TIRAP and facilitating the recruitment of MyD88 signaling adaptor to initiate TLR signaling (123). Several studies have shown a crosstalk between complement and TLR-4. It is known that TLR-4 activation by LPS can induce C5aR up-regulation on hepatocytes mediated by IL-6 (124). Another, immune-modulatory function of C5a is to reduce the production of IL-12 family cytokines by mouse inflammatory macrophages stimulated by TLR-4 ligands (125). In turn, this resulted in regulation of Th1 cells polarization and a limitation of their expansion. A synergy between C5a and TLR ligands on mouse DC stimulation was found upon stimulation of these cells with a fusion protein composed of C5a and an endogenous ligand for TLR-4 (extra domain A) and an antigen (126). This induced strong antigen-specific T cell responses *in vivo*, without production of immunosuppressive molecules. In humans, the C5a-mediated immature DC stimulation appears more complex. C5a increases the cytokines production in immature DCs, but upon TLR-4 stimulation, C5a inhibits the production of IL-12, IL-23, and TNFα, thus having an anti-inflammatory role (127). These results emphasize that the effects of anaphylatoxins on immune response depend on the crosstalk not only with TLRs but also with other receptors.

## Complement and Adaptive Immunity

Discoveries over five decades have shown that the complement system plays an important role not only in the inflammation but also in the adaptive immunity.

### The complement system and its interplay with B cells

**Figure 6:**
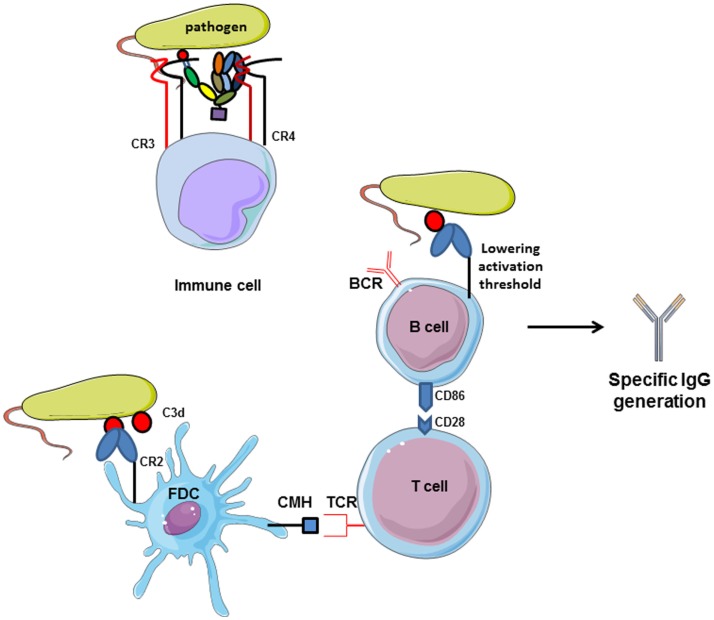
**Complement receptors implication in adaptive immunity**. CR2 activation by interaction with C3d-opsonized antigen on follicular dendritic cells increases CMH expression and allows antigen presentation to TCR. Then, costimulatory molecules are expressed and T lymphocytes help in memory B cells maturation in germinal centers. Moreover, C3d/CR2 interaction lowers the activation threshold of B cells and increases BCR signaling activity. Cumulated, C3d/CR2 interaction induces specific IgG generation by B cells, and C3d works as a natural adjuvant. CR3 and CR4, expressed on immune cells.

The relationship between complement and B cells has already been demonstrated 40 years ago *in vitro* and using *in vivo* models. C3 plays important role in antibody generation by B cells (Figure 6). In case of C3 depletion [by a structural analog of C3 from snake venom (CVF), which is capable to bind to FB and to activate complement in fluid phase], the humoral immunity toward certain thymus-dependent antigens and the lymphocyte cooperation was impaired (128, 129). These and other early studies demonstrated that complement binds and localizes foreign antigens within sites where the lymphocytes response takes place. B cells express complement receptor CR2 (CD21), which interacts with C3d and iC3b on the surface of the antigen and forms also a co-receptor complex with CD19 and CD81 (130). Thus, the complex C3d:CR2 induces an increase of B cell receptor (BCR) signaling in the presence of C3d-opsonized antigen on B cell surface (131). *In vivo*, C3 is required for the induction and maintenance of memory cells of the B cell lineage within the microenvironment of germinal centers (GCs), where B cells encounter antigen–antibody–C3 complexes on the surfaces of follicular dendritic cells (FDCs) (132). When C3d-opsonized antigen binds to CR2 on FDCs, they can present the antigen in the GC and induce effector and memory B cells (133). This underlines the importance of CR2 expression on FDCs and B cell surface for the generation of antigen-specific IgG. C3d serves as a molecular adjuvant by lowering the threshold for B cell activation by 1000 to 10,000 times (134).

The human complement regulator of the CP C4b binding protein (C4BP) has functions extending beyond the dissociation of the classical C3 convertase and serving as cofactor for FI for C4b inactivation. It binds directly to the costimulatory protein CD40 on human B cells at a site that differs from that used by the CD40 ligand. C4BP induces proliferation, the up-regulation of CD54 and CD86 expressions, and IL4-dependent IgE isotype switching in normal B cells. These observations suggest that C4BP is an activating ligand for CD40 and establish another interface between complement and B cell activation (135).

The auto-reactivity of B cell is also tightly regulated by complement. C4 has been demonstrated to be essential to maintain peripheral B cell tolerance (136). Deficiency in C4 promotes the emergence of autoreactive B cells during the maturation in the GCs. These results could be explained by a lack in the clearance of apoptotic cells in GCs that leads to an impairment in host antigen presentation by APC, essential for the education and the peripheral B cell tolerance.

The complement system can contribute to autoimmune diseases by decreasing the threshold for B cell activation. For example, in a collagen-induced arthritis (CIA) mouse model, C3 depletion delays and decreases severity of the disease (137). Complement via C3d plays a key role in B cell function, and C3d antigen can break anergy in autoreactive B cells (138). Complement can modify the antigen-specific B cell response in experimental autoimmune encephalomyelitis (EAE) and possibly in multiple sclerosis (MS). In EAE mouse model, consumption of complement, using CVF, significantly attenuates clinical and histological EAE (139). The authors suggest that complement breaks the anergy of autoreactive B cells, leading to autoantigen-specific IgG production, while the total IgG response remained unaffected.

### The complement system and its interplay with T cells

**Figure 7:**
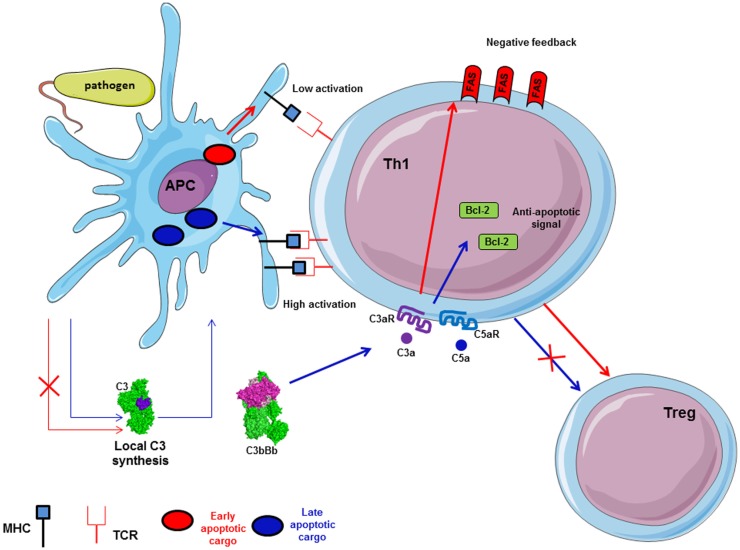
**Local synthesis of C3 by antigen presenting cells (APC) is implicated in antigen-specific T cell response**. C3 synthesis by APC in case of pathogen internalization induces late apoptotic cargo and allows high expression of MHC on APC surface. Thus, it participates in antigen-specific T cell proliferation and Th1 generation. Local C3 and C5 generate C3a and C5a, which induce up-regulation of Bcl-2 and down-regulation of FAS expression via C3aR and C5aR to facilitate T cell proliferation. Moreover, C3a/C3aR and C5a/C5aR signaling pathways activation promotes Th1 generation and avoid Treg differentiation.

The importance of complement for the survival of resting T cells has been described above. Upon infection with a pathogen, T cell proliferation and differentiation are controlled by APC and their microenvironment. Complement is able to polarize T cells and participates to the induction and effector phase, as well as to the contraction phase of the T cell response (140–142). In fact, during inflammation, anaphylatoxins C3a and C5a are able to bind their corresponding receptors expressed on the T cells and APC surface, leading to cytokine production by these cells (27) (Figure 7). Local C3 synthesis by DC is necessary to induce T cell activation and Th1 response (28). C3 deficiency was shown to accelerate the fusion of the apoptotic cargo with lysosomes and led to impaired antigen-specific T cell proliferation *in vitro* and *in vivo* (143). Moreover, C3a/C3aR is responsible for up-regulation of the anti-apoptotic Bcl2 and down-regulation of the pro-apoptotic molecule FAS during infection on myeloid and lymphoid cells, inducing immune cells survival and proliferation (144). The absence of C3aR and C5aR stimulation during T cell activation induces Treg development (145, 146). Thus, C3-deficient patients, who cannot produce C3a and C3b, present a lack of Th1 response whereas Th2 response remains normal (147, 148).

**Figure 8:**
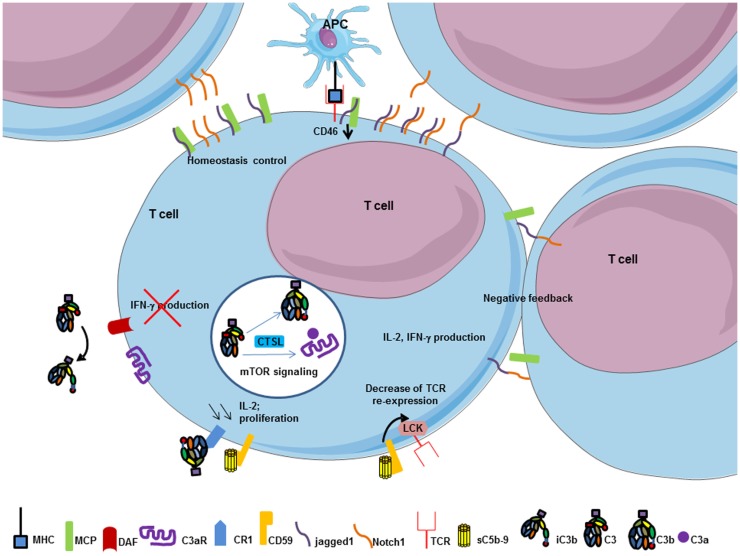
**T cell response is modulated by complement components**. MHC/TCR interaction between APC and T cell decrease CD46 expression on T cell and allows *cis* interaction between jagged1 and Notch-1 on T cell surface to promote T cell proliferation, IL-2, and IFN-γ production. Thereafter, *trans* interaction between jagged1 and Notch-1, and CD46 work as negative feedback to control T cell homeostasis. Soluble C5b–9 and CR1 regulate T cell activation. Interaction between soluble form of C5b–9 and its specific inhibitor CD59 on T cells decrease TCR re-expression after its internalization to limit T cell activation by transmitting a signal via Lck. CR1 activation by iC3b decreases IL-2 synthesis and proliferation of T cell to promote a negative feedback of T cell activation. CD55 engagement on T cells negatively regulates Th1 induction cells by inhibiting IFN-γ production. Intracellular C3 in T cell is cleaved by CTSL and promotes C3b and C3a intracellular generation. Interaction between C3a and C3aR induces mTOR signaling and survival signal of the immune cell.

The complement regulator CD46 plays an important role in the regulation of T cells (149) (Figure 8). CD46 has different isoforms affecting its cytoplasmic tail and resulting from alternative splicing. Different isoforms are expressed in different organs. CD46 engagement on CD4^+^ T cells promotes the effector potential of Th1 cells (150). As IL-2 accumulates, it switches cells toward a regulatory phenotype, attenuating IL-2 production and upregulating IL-10. The interaction of the CD46 cytoplasmic tail with the serine–threonine kinase SPAK plays an important role in this process. The γδ T cells express an alternative CD46 isoform and thus are unable to switch from IL2 to IL10 production. The Treg express different CD46 cytoplasmic tails, as compared to Th1 cells. Therefore, CD46 uses distinct mechanisms to regulate different T cell subsets during an immune response. Recently, the Notch family member Jagged1, which is expressed on T cells, has been identified as a new natural ligand of CD46 (147). Jagged1 binds Notch-1 and this interaction is responsible for cellular activation and proliferation. The *cis* interaction (on the same cell) of CD46 and Jagged1 leads to a competition with Notch, thus controlling the homeostasis of naïve T cells and inhibiting their activation. In the case of TCR stimulation, CD46 is down-regulated, which allows Notch-1 and Jagged1 to bind in *cis* and in *trans* (from two different cells). This induces Th1 proliferation and polarization, leading to IFN-γ and IL2 induction. Interestingly, CD46-deficient patients are unable to produce IFN-γ and have a lack of Th1 response, whereas Th2 response remains normal. Taken together, these results suggest that the expression of CD46 is necessary to induce a Th1 response. Then, CD46 plays a negative feedback when proliferation leads to T cell/T cell contact limiting the expansion of Th1 cells and allows a contraction phase. The binding site for Jagged1 on CD46 has been mapped to the first two CCP domains (147). There is no overlap between this binding site and the C3b binding site, which is located in the CCP3 and 4.

CD55 (decay accelerating factor, DAF) also plays a role in the establishment of the adaptive immune response. In addition to control complement activation, experiments on CD55 deficient mice showed an enhanced Th1 response with a hypersecretion of IFN-γ (151, 152). This may be explained by the overactivation of complement leading to strong local anaphylatoxin production (153).

CD35 and CD59 also participate in T cell regulation. Recent study showed that CD59 is able to modulate T cell activation by transmitting a signal via Lck to TCR/CD3ζ. A knock-down of Lck accelerated the re-expression of CD3 at the cell surface (154). Engagement of CD35 (CR1) on T cells reduces their rate of proliferation and IL-2 secretion (155). Reduction of the expression of CD46 on activated T cells may lead to local complement overactivation, thus generating larger amount of C3b, iC3b, and C5b–9. Therefore, binding of C3b and iC3b to CD35 and C5b–9 to CD59 could contribute to the negative feedback controlling Th1 expansion (149).

In addition, intracellular mechanisms of sensing C3b-opsonized pathogens have recently been described (156). Pathogens that cross the cell membrane, with covalently attached complement C3 on their surface, activate mitochondrial antiviral signaling. This mechanism would represent an autonomous immunity of the cells against non-enveloped viruses and cytosolic bacteria, by inducing signaling pathways, such as NF-kB, IRF3/5/7, and AP-1, leading to pro-inflammatory cytokines production (156).

All these examples illustrate the importance of complement for the mounting of a successful immune response. Therefore, this cascade should not only be considered simply as a humoral factor, mediating innate immunity and inflammation, but also as a potent regulator of cells functions in the adaptive immunity.

## Strategies of the Pathogens to Evade the Complement System

**Figure 9:**
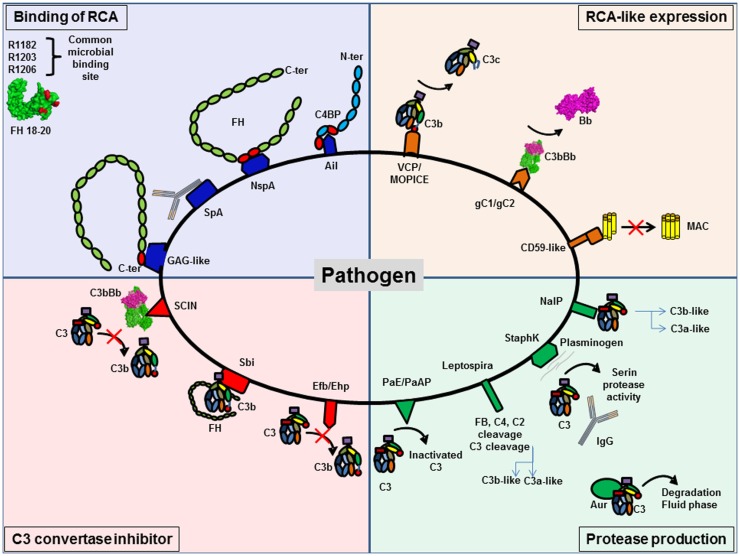
**Pathogens are able to protect themselves from complement activation**. Pathogens have developed different strategies to inhibit complement activation. They can be classified in four different groups. Several pathogens are able to bind regulators of complement activation (RCA), such as FH and C4BP, to decrease C3 deposition. RCA-like expression allows pathogens to block complement activation without the need to recruit complement regulator. Synthesis of proteases specifically against complement proteins degrades complement components. The last group is pathogens able to express C3 convertase inhibitors.

Complement system works as a first line of defense and allows in many cases to avoid infections. Nevertheless, the evolution of the pathogens resulted in elaboration of evasion strategies against complement attack. These mechanisms of complement evasion can be classified in different groups (157) (Figure 9).

### Binding of host complement regulators

Binding of host complement regulators on their membrane allows pathogens to inactivate complement. Bacteria, viruses, fungi, and parasites have been shown to bind high levels of efficient complement regulators such as FH, factor H-like 1 (CFHL-1), and C4 binding protein (C4BP) (158). Recruitment of these regulators can accelerate the decay of the C3 convertase and provide cofactors for FI, which cleaves C4b and C3b, thus protecting the pathogen against complement attack.

To bind fluid phase complement regulators, pathogens express specific molecular platforms with high affinity, such as PorA in *Neisseria meningitidis* (159), filamentous hemagglutinin in *Bordetella pertussis* (160), Ail in *Yersinia pseudotuberculosis* (161), factor H binding protein (fHbp) and neisserial surface protein A (NspA) in *N. meningitidis* (162), and M protein family in group A streptococci (163).

Factor H binds to pathogens by a “common microbial binding site,” located in its C-terminal domain CCP20. This domain binds to heparin and other GAGs on the host cell surface, but the binding sites for GAG and for pathogens surface proteins are not identical (164, 165). Three key amino acids (R1182, R1203, R1206) are critical for this “common microbial binding site” for at least seven Gram-negative and Gram-positive pathogens (165). In addition, the microbial proteins enhanced binding of FH19–20 to C3b, forming a triple complex, and leading to a more efficient complement inactivation on the pathogen surface. This is a unique example of convergent evolution, resulting in enhanced immune evasion of important pathogens via utilization of a “superevasion site” (165).

In addition to CCP 19–20, pathogens trap FH by binding to CCP6–8. *Neisseria* species bind this region of FH. Por1A from *Neisseria gonorrhoeae*, interacts with CCP 6, in addition to CCP 19–20, to escape from complement attack (166). Also, fHbp and NspA from *N. meningitidis* can recruit FH by binding to CCP 6–7 (162, 167). Study of the structure of fHbp by mutagenesis revealed that fHbp binding site for CCP 6–7 overlaps with sucrose octasulfate, a GAG analog. FhbB from *Treponema denticola* binds FH by a similar mechanism. Study of FH/FhbB interaction by site-directed mutagenesis revealed a binding site localized in CCP7 of FH (168).

*Staphylococcus aureus* has developed a different strategy to prevent complement initiation. Staphylococcal protein A (SpA) recognizes the Fc portion of Igs with high affinity and hide the binding site for C1q, thus blocking the initiation of the CP (169). The outer membrane protein SdrE binds FH and C4BP to *S. aureus* (170, 171). Together, these findings suggest that *S. aureus* is able to inhibit the three complement pathways.

### Expression of complement regulators-like proteins

Expression of complement regulators-like proteins can contribute to pathogens camouflage. Some viruses, such as poxvirus, have found ways to produce soluble proteins that closely mimic the structure and function of host regulators, such as vaccinia virus, which produce VCP, a protein similar to CD55 and MCP. Thus, these viruses inhibit both CP and AP, dissociating C3 convertases formed on C3b and C4b. Monkeypox virus produces a complement regulator-like protein, MOPICE, which is able to bind human C3b and C4b and serves as cofactor for FI (172). MOPICE is considered as a virulence factor of this virus, since it is expressed in the more virulent strains from Central Africa and absent in the less virulent pathogens from West Africa. Another example of complement regulators-like activity comes from Herpes viruses, which express transmembrane gC1 and gC2 glycoproteins able to bind C3b and to specifically accelerate the decay of the AP C3 convertase (173). *Borrelia burgdorferi* produces a CD59-like protein, which has affinity for C8b and C9 and avoids MAC formation (157).

### Production of proteases that degrade complement

Production of proteases that degrade complement proteins is frequently observed in bacteria. Indeed, *Pseudomonas* produce *Pseudomonas* elastase (PaE) and alkaline protease (PaAP) that cleaves immunoglobulins and C1q, thus preventing activation of the CP (174). In addition, these proteases are able to inactivate C3 into a non-functional fragment and inhibit complement activation (174). *Leptospira* strains are able to cleave directly complement proteins and inhibit the three complement pathways. *Leptospira* produced metalloproteases may not only cleave C3 but also FB, C4, and C2 (175). Generated C3 degradation products differ from those obtained by FI, suggesting that they would not result in opsonization and phagocytosis. *S. aureus* is also able to produce distinct proteolytic enzymes against complement proteins. Staphylokinase can form a complex with plasminogen, resulting in a serine protease activity efficient against C3 and IgG. Moreover, plasmin is able to bind the surface of *S. aureus* and inhibits the binding of IgG, C3b, and iC3b and blocks the effect of opsonization (176). This enzyme activity leads to the CP inhibition. *S. aureus* produces four important proteases: cysteine proteases staphopain A and B, serine protease V8, and metalloproteinase Aur. These proteases lead to a drastic decrease in the hemolytic activity of serum and are efficient against the three pathways (177). Aur is responsible for the consumption of C3 in the fluid phase (178). *N. meningitidis* is able to produce a protease NalP, which cleaves C3. This degradation produces two fragments, a shorter C3a-like and a longer C3b-like, degraded by host serum and leading to a decreased C3b deposition on the bacterial surface (179). *N. meningitidis* is able to inhibit the CP by capsular oligosaccharides, which represents a virulence factor of meningococcal infections (180). Capsular oligosaccharides interfere with engagement of C1q by IgG Fc, and lead to decreased C4b deposition and inhibition of CP activation.

Viruses can also produce C3b cleaving enzymes. Nipah virus particles carry a FI-like protease activity able to cleave and inactivate C3b, using FH and CR1, but not CD46, as cofactors (181). These data help to explain how an enveloped virus such as Nipah virus can infect and disseminate through body fluids that are rich in complement.

### Production of inhibitors of C3 convertase

Production of inhibitors of C3 convertase has been observed in a few cases. *S. aureus* produces extracellular fibrinogen-binding protein (Efb), which inhibits the conversion of C3 to C3b by its affinity for the thioesther domain (TED). It binds to the TED domain on an area, shared by FH and CR2 binding sites (182–185). Efb acts as a C3 convertase inhibitor by blocking C3b formation and inhibits the opsonophagocytosis by granulocytes (186). Ehp (also known as Ecb), a homologous protein of Efb, binds two molecules of C3b and works as an efficient inhibitor of the alternative C3 convertase (187). Thus, it leads to a decrease of the activation of the C5 convertase and of the resulting C5a level. Surface immunoglobulin-binding protein (Sbi) forms a tripartite complex with C3b and functional FH that potentiates inhibition of complement activation (188). Efb and Sbi are able to recruit human plasminogen after binding to C3/C3b. Plasminogen is converted to plasmin by bacterial staphylokinase or by host-specific urokinase-type plasminogen activator and degrades C3 and C3b in the local microenvironment, thus protecting *S. aureus* (184, 189). Of note, the tripartite complex formed by C3, plasminogen, and Efb-C is more efficient than the one with Sbi, probably due to the higher affinity of C3d to Efb than Sbi, (184). The action of these molecules prevents the activity of the C3 and C5, as shown by a decreased C5a generation (190).

Contrary to Efb, staphylococcal complement inhibitor family (SCIN-A, SCIN-B, SCIN-C), can bind C3b in two distinct regions with a primary site at domain MG8 (191–194). By competing with FB and FH, SCIN is able to block the formation of alternative C3 convertase on one side, and to block the generation of iC3b that could induce phagocytosis on the other side. Moreover, SCIN binds and stabilizes the C3 convertase C3bBb, blocks the decay acceleration, and inhibits the cleavage of C3 into C3b. Thus, it avoids opsonization and complement activation in the same time (193).

Targeting binding sites for complement inhibitors on bacterial surfaces and complement-degrading proteases with vaccine-induced antibodies may be used as a strategy for novel vaccines designed to prevent complement escape. On the other hand, some bacterial proteins with anti-complement activity, such as Efb and SCIN, represent potential novel therapeutics, able to control undesired complement activation and tissue injury in multiple non-infectious diseases (195, 196). It is important to consider that by blocking C3 convertase formation, these inhibitors will avoid opsonophagocytosis and, by such, increase the risk of infection in treated patients (197). The increasing knowledge of the complement evasion strategies of pathogens provides novel insights for more efficient vaccine development and for designing novel therapeutic complement inhibitors.

## Complement and Non-Infectious Diseases

**Figure 10:**
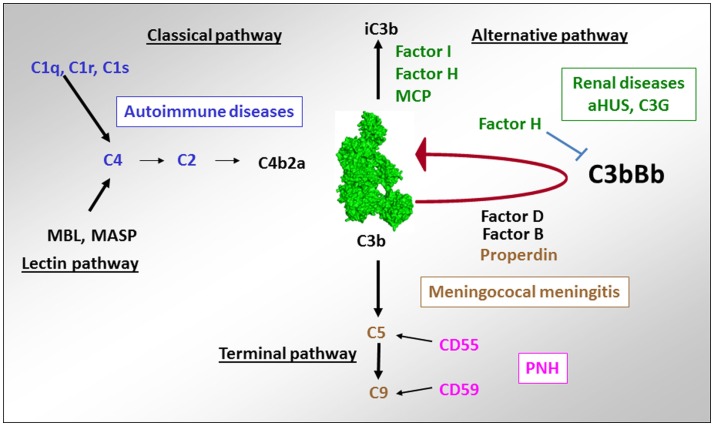
**Implication of complement deficiencies in pathologies**. Deficiencies of the components of the CP C1q, C1r, C1s, C2, and C4 are associated with autoimmunity. Lack of regulators of the AP FH, CD46, and FI (as well as overactivation of the components of the C3 convertase C3 and FB) is linked to aHUS and C3G. Deficiencies of the terminal complement components C5, C6, C7, C8, and C9 as well as of the only positive regulator of complement – properdin is susceptibility factors to development of meningococcal meningitis. Lack of expression of the regulators of the C3/C5 convertase CD55 and the terminal pathway CD59 on erythrocytes are a cause of red cell lysis in paroxysmal nocturnal hemoglobinuria (PNH).

The importance of the complement system in physiology is illustrated by severe and life threatening diseases, occurring in case of inefficient or exuberant complement activity (Figure 10). Abnormal complement activity is associated with a large number of inflammatory, autoimmune, thrombotic, and age-related diseases. The examples of systemic lupus erythematosus (SLE), atypical hemolytic uremic syndrome (aHUS), C3 glomerulopathies (C3G), age-related macular degeneration (AMD), and cancer are treated here in more details, but these are just few among a large list of diseases, including also the paroxysmal nocturnal hemoglobinuria (PNH), graft rejection after transplantation, ischemia/reperfusion injury, Alzheimer and Parkinson diseases, etc.

## Diseases Associated with Deficient Complement Activation

### Systemic lupus erythematosus

Deficiency of components of the classical complement pathway C1q, C1r, C1s, and C4 are very rare but are strongly associated with autoimmune manifestations. The autoimmune disease SLE is characterized by symptoms ranging from skin rashes, chronic fatigue, and arthritis to the more severe glomerulonephritis, serositis, and neurological involvement (198). C1q is the strongest genetic susceptibility factor for SLE, since over 90% of the individuals homozygous for mutations in this protein develop lupus-like symptoms (199). The mutations can cause quantitative deficiency (199) or functional abnormalities (13). A functional abnormality of C1q, due to a mutation in the C1r2C1s2 binding site, resulted in an inability of C1 complex formation and presence of free C1q in the patient serum. The strong association of the mutations of the CP proteins with autoimmunity can be explained by the “waste disposal” hypothesis of Walport (17), suggesting that lack of C1q opsonization and CP activity perturbs the immunologically silent uptake of apoptotic cells and debris, thus resulting in an immune response against self-antigens.

Alteration of the function of C1q can be also induced by presence of autoantibodies against C1q, which are frequently found in SLE, especially in patients with lupus nephritis (200). These antibodies bind to the collagenous tail of C1q (201) or the globular domain (202). These antibodies amplify the effect of C1q-containing immune complexes in the kidneys (203, 204), bind to C1q on early-apoptotic cells, and enhance complement activation (205).

Impaired clearance of apoptotic cells, induced by lack of C1q or inefficient complement activation due to lack of C1r, C1s, C2, and C4 are associated with SLE. At the same time, complement overactivation, due to the presence of anti-C1q antibodies or complement activating immune complexes in the circulation and in kidney glomeruli, is also leading to the same disease, albeit by a different mechanism. Therefore, both lack of activation and a too strong activation of the CP can be linked to autoimmunity.

### Diseases associated with complement overactivation

Inherited and acquired quantitative and functional deficiency of the regulators of the alternative complement pathway FH, FI, and CD46 as well as abnormalities inducing overactivation of the AP C3 convertase are associated with rare renal diseases like aHUS (206, 207) and C3G (208, 209).

### Atypical hemolytic uremic syndrome

Atypical hemolytic uremic syndrome is a renal thrombotic microangiopathy disease, characterized by glomerular microvascular endothelial cells activation and damage, leading to microthrombi formation and mechanical hemolysis (206). Within the last decade, complement AP dysregulation has emerged as the cause of aHUS (207, 210). In this disease, complement induces glomerular endothelium damage, secondary to unrestricted complement activation with C5b–9 deposits on the endothelial cells surface. A loss of regulation is a result of mutations in FH, FI, or CD46 genes, found in ~50% of the cases. FH mutations are frequently located in the C-terminal domains and result in reduced capacity of FH to bind C3b and the GAG of the cell membrane (211–213). Mutations in FI impair its catalytic activity (214, 215). Complement overactivation in 10% of the cases is due to mutations in the components of the C3 convertase C3 and FB, forming a more potent convertase and/or a converatase resistant to decay by the regulators (216–221). In addition to mutations in complement genes, autoantibodies against FH, leading to an acquired FH functional deficiency, have been reported in aHUS patients (222–224). The binding epitopes of these autoantibodies are localized to the C-terminal recognition region of FH, which represents a hot spot for aHUS mutations (225).

Altogether complement-associated abnormalities are found in about 60% of the aHUS patients (226, 227). At present, over 200 distinct mutations have been reported in aHUS patients (207). All reported mutations were heterozygous, except in 15 patients (mostly from consanguineous families) with homozygous FH or CD46 deficiency (228). In a particular form of aHUS, mutations were found in DGKE – a gene outside of complement, involved in the protein kinase C signaling pathway (229). Its deficiency induces permanent pro-coagulant phenotype of the endothelial cells (230). aHUS has an incomplete penetrance among the mutation carriers and a triggering event (infection, pregnancy, etc.) and additional genetic predisposing factors (particular at risk haplotypes in FH and CD46) are needed to induce the disease (220, 231, 232). The hemolysis, which accompanies the disease onset, can serve as a secondary hit for the aHUS development, since released heme activates the alternative complement pathway in the fluid phase and on the endothelial cells surface (233, 234). The availability of the first effective anti-complement therapeutic drug, eculizumab, has changed dramatically the outcome of this rare kidney disease.

### C3 glomerulopathies

C3 glomerulopathies are rare chronic kidney diseases, characterized by predominant C3 deposits in glomeruli, in particular, in the mesangium and along the glomerular basement membrane, frequently associated with mesangial cells proliferation. According to the pattern of C3 deposits observed by immunofluorescence and electronic microscopy, two subtypes of C3G are described – dense deposit disease (DDD) and C3 glomerulonephritis (C3GN) (208). The pathogenic process in C3G is presumed to be due to uncontrolled C3 and/or C5 convertases activation, leading to C3 deposits and intra-glomerular inflammation. Autoantibodies targeting the AP C3 convertase, named C3 nephritic factor, are present in more than 50% of C3G patients (235–237). Few genetic abnormalities have been identified. These include mutations of complement genes, coding for C3 (1 case) (238) and the regulator FH (<20 mutations) (237, 239). Mutations affecting *FH* gene result more frequently a protein deficiency in the plasma. More recently, mutations in the *CFHR5* gene were reported, as well as rearrangements and copy number variations in the *CFHR* gene cluster (240–242). A particular form of C3GN is the CFHR5 nephropathy, characterized by a genetic defect in CFHR5, rendering it a strong competitor of FH. It is found in patients from of Cypriot descent (241, 243).

In C3G, genetic abnormalities affect the same genes as in aHUS, with a lesser frequency compared to aHUS, affecting only about 20% of the patients (209, 237). An interesting observation is that the mutations in FH and in FI, which are associated with C3G, are frequently the same, as the ones found in aHUS. They induce either complete or partial FH deficiency or functional defects in the N-terminal part of the protein (237). Mutations in the membrane anchoring C-terminal part of FH are found frequently in aHUS and nearly not in C3G. Therefore, the mutation itself (particularly the deficiencies of FH and FI and the mutations in the N-terminal part of FH) may not be sufficient to determine the type of the renal injury. The triggers of the AP dysregulation remain undetermined.

C3 nephritic factor and FH or FI mutations, inducing an overactivation of the AP are found also in patients with another type of membranoproliferative glomerulonephritis, associated with immunoglobulin deposits in the kidney – MPGN type I (237). Therefore, the role of the AP has to be considered even in diseases, for which CP activation is expected.

### Contribution of complement in diseases, not associated with complement defects

#### Age-Related Macular Degeneration

**Figure 11:**
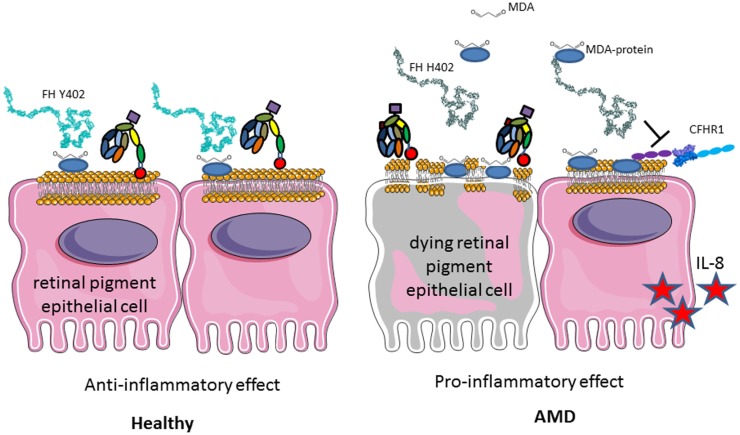
**Role of factor H Y402H polymorphism in age-related macular degeneration**. FH binds to GAG, oxidized lipids, including malondialdehyde via CCP7 on the membrane of injured retinal epithelial (RPE) cells and protects them from complement attack. H402 variant has a weaker affinity to these products of the oxidative stress and protects less well the cells surface. Deposited C3b is not inactivated to iC3b and promotes complement activation. Less iC3b is generated, which contributes to the silent clearance of the injured cells. RPE cells are activated, secrete pro-inflammatory cytokines, and activate macrophages in their microenvironment. This chronic inflammation predisposes to AMD development with aging.

A particular polymorphism of FH (Y402H) is strongly associated with development of AMD, which is the first cause of blindness in the developed countries (244–247). The loss of central vision is associated with loss of photoreceptors and drusen formation in the retina (248). This is due to the constant exposure to light, smoking, the high-metabolic rate in the eye, and its particular sensibility to oxidative stress. Oxidized lipids and malondialdehyde are generated and if not properly handled by FH, they induce complement activation (249, 250) (Figure 11). FH binding to oxidized epitopes on altered or dying cells leads to inactivation of C3b to an anti-inflammatory fragment iC3b. Moreover, FH attenuates malondialdehyde-induced IL-8 production by macrophages and retinal pigment epithelial cells and decreases the expression of genes involved in macrophage infiltration, inflammation, and neovascularization in the eye. At risk, FH variant H402 has a weaker capacity to interact with oxidized lipids and malondialdehyde in the drusen, thus allowing constant background complement activation, leading to retinal epithelial cells damage and macrophages activation (249, 250). These results explain the strong genetic association of the H402 with the risk of AMD. Increased risk for AMD is conferred also by polymorphisms in several other genes of the alternative complement pathway, including FI, C3, C2/FB, and C9 (251).

Genetic analyses revealed that the deletion of CFHR1 and CFHR3, which is a polymorphism in the normal population with varying frequency depending on the ethnicity between 2 and 20% (252), is a protective factor against development of AMD (253). CFHR1 and CFHR3 are natural deregulators of FH, competing with it for cell surface binding (241, 254, 255). Therefore, their absence will increase the local binding of FH to oxidized surfaces, leading to a better protection, thus explaining the protective effect of this deletion in AMD.

### Cancer

Complement has been considered since a long time as an immune surveillance system against cancer, because complement is activated on the surface of tumor cells. Nevertheless, tumor cells develop inhibitory mechanisms for the terminal steps of the complement cascade, thus preventing complement-mediated cytotoxicity. Surprisingly, recent studies demonstrated that complement activation within the tumor microenvironment can promote tumor growth. Complement activation may support chronic inflammation, promote an immunosuppressive microenvironment, induce angiogenesis, and activate cancer-related signaling pathways. The mechanisms of these phenomena are not fully understood. Prolonged complement activation supports chronic inflammation, promotes an immunosuppressive microenvironment, induces angiogenesis, and activates cancer-related signaling pathways.

**Figure 12:**
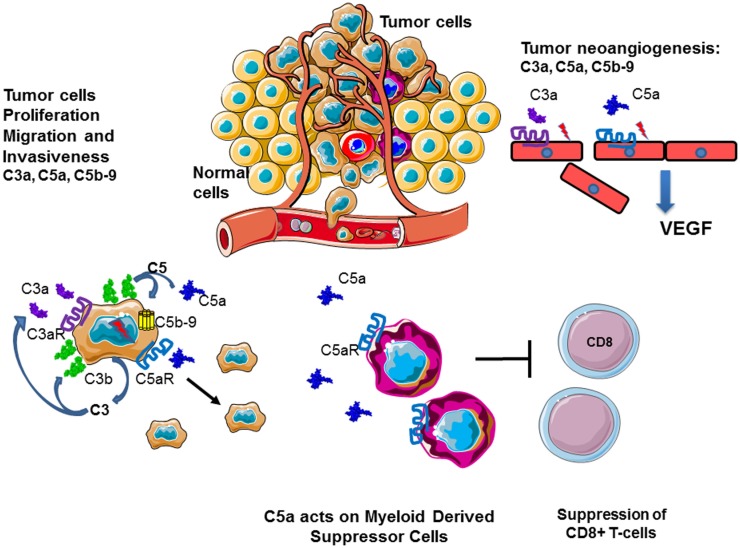
**Role of complement in cancer**. Complement plays an important role in the chronic inflammation and tumor development. Tumor cells produce complement components and generate C3a, C5a, and C5b–9 in their microenvironment. This result in autocrine tumor cells stimulation, leading to proliferation, migration, and invasiveness. C5a stimulates MDSC, which dampen the immune response, suppressing cytotoxic T cells, and stimulate Treg. C3a, C5a, and C5b–9 promote angiogenesis, helping in tumor nutrient support and dissemination.

Several lines of evidence indicate a role for molecules of the complement system in tumor growth and metastasis, (Figure 12). C3, C4, or C5aR deficiencies prevent tumor growth in mice, potentially via inhibition of the CP and the generation of C5a, which has a potent inflammatory potential. In mouse models, the presence of C5a in the tumor microenvironment enhances tumor growth by recruitment of MDSC and increasing T cell-directed suppressive abilities (103, 256, 257). In a breast cancer model, C5aR facilitated metastasis in the lungs through different immune mechanisms in the metastatic niche, including the suppression of effector CD8(+) and CD4(+) T cell responses, the recruitment of immature myeloid cells and the generation of Tregs and a Th2-oriented response (258).

Cancer cells also secrete complement proteins that stimulate tumor growth upon activation via a direct autocrine effect through C3aR and C5aR signaling (256). In patients with ovarian or lung cancer, higher tumoral C3 or C5aR mRNA levels were associated with decreased overall survival. In addition, patients with non-small cell lung cancer have elevated C5a plasma levels (257).

C3a and C5a seem to have opposing effects during tumor development and in case of anti-tumor radiotherapy. While C3a and especially C5a promote tumor growth, radiotherapy-induced tumor cell death and transient local complement activation with production of C3a and C5a (259). The latter appeared crucial to the tumor response to radiotherapy and concomitant stimulation of tumor-specific immunity.

Overexpression of FH has been described in non-small cell lung cancer cell lines and on non-small cell lung cancer biopsies (but not in small cell lung carcinoma and carcinoid cell lines) (260, 261), in bladder tumor cells (262), in cutaneous squamous cell carcinoma (cSCC) and cell lines (263), and in hepatocellular carcinoma tumors (264). Low titer anti-FH antibodies were also found in sera from patients with non-small cell lung cancer (265). Recent studies demonstrated that FH binds to pentraxin 3 (PTX3) in the tumor microenvironment, thus preventing local complement overactivation and generation of pro-tumorigenic C5a (266). Hence, deficiency of PTX3 accelerated tumor development in mouse models of drug-induced cancerogenesis. FI was also suggested to be associated with tumor development of cSCC (267). These results provided evidence for a potential role of FH and FI in the cancer development, but the mechanism of action are still unknown.

These examples clearly indicate that complement is indispensable immunosurveillance system, which needs to function with the right force when and where is needed. Therefore, therapeutic strategies are needed to adjust the level of complement activation in pathological conditions.

## Complement as a Therapeutic Target

Since C1q is produced by the myeloid cells, a bone marrow transplantation can overcome this deficiency. Such therapy was efficiently applied to a patient with SLE, resulting in normalization of C1q levels and improvement of the clinical status (268). Deficiency of C1INH in hereditary and autoimmune angioedema (which is not a complement-mediated disease, but induced by excessive production of bradykinin, which is a potent vasodilator) is efficiently treated by plasma derived or recombinant C1INH (269). Alternatively, missing soluble complement components can be introduced to the body by plasma exchange. This was successfully achieved for C1q (270) as well as for FH and FI deficiency in aHUS. Plasma therapy was the first line treatment for aHUS before the development of anti-C5 blocking antibody eculizumab (271, 272). Eculizumab binds to C5 and prevents its entry into the C5 convertase, thus blocking completely the generation of C5a and the formation of MAC (273). This therapeutic is approved for use in PNH and aHUS and gives excellent results (274, 275). Currently, clinical trials are ongoing for many different diseases, where complement is overactivated. Novel inhibitors are under development to block complement at different levels. Particular interest is focused on the blockers of complement at the level of C3 and the C3 convertase. C3b binding peptide compstatin and its derivatives prevent the entry of C3 into the C3 convertase (276, 277). Plasma derived or recombinant FH and recombinant soluble CR1 dissociate the alternative C3 convertase and serve as cofactors for FI (278). Targeted inhibitors like TT30 (FH CCP1–5:CR2) or mini FH (CCP1–4:CCP19–20) bring the regulatory N-terminal domains to the cell membrane, where they are needed to control complement activation (279–282).

## Conclusion

Complement plays a central role in the homeostasis and the installation of the adaptive innate immune response. By its supporting role in the clearance of apoptotic and necrotic cells, and its importance in the polarization of T lymphocytes and the humoral response by the cooperation with the B-lymphocytes, complement represents a real keystone of the immune system. This crucial role for the correct functioning of the organism is illustrated by the fact that both complement deficiencies and complement overactivation are associated with severe and life threatening diseases. The improvement of our understanding of the role of complement in health and disease opens up the possibility to use complement modulating drugs in the clinical practice.

## Conflict of Interest Statement

The authors declare that the research was conducted in the absence of any commercial or financial relationships that could be construed as a potential conflict of interest.
